# Nationwide emergency department visits for pediatric traumatic spinal cord injury in the United States, 2016–2020

**DOI:** 10.3389/fneur.2023.1264589

**Published:** 2023-11-10

**Authors:** James A. G. Crispo, Lisa J. W. Liu, Vanessa K. Noonan, Nancy P. Thorogood, Brian K. Kwon, Marcel F. Dvorak, Dylan Thibault, Allison W. Willis, Jacquelyn J. Cragg

**Affiliations:** ^1^Collaboration for Outcomes Research and Evaluation (CORE), Faculty of Pharmaceutical Sciences, University of British Columbia, Vancouver, BC, Canada; ^2^Human Sciences Division, NOSM University, Sudbury, ON, Canada; ^3^International Collaboration on Repair Discoveries (ICORD), University of British Columbia, Vancouver, BC, Canada; ^4^Praxis Spinal Cord Institute, Vancouver, BC, Canada; ^5^Department of Orthopaedics, University of British Columbia, Vancouver, BC, Canada; ^6^Department of Neurology, University of Pennsylvania Perelman School of Medicine, Philadelphia, PA, United States; ^7^Department of Biostatistics, Epidemiology and Informatics, University of Pennsylvania Perelman School of Medicine, Philadelphia, PA, United States

**Keywords:** spinal cord injury, epidemiology, pediatric, emergency department, sports

## Abstract

**Introduction:**

Traumatic spinal cord injury (tSCI) is a debilitating neurological condition resulting in lifelong disability for many individuals. The primary objectives of our study were to describe national trends in incident emergency department (ED) visits for tSCI among children (less than 21 years) in the United States, and to determine the proportion of visits that resulted in immediate hospitalization each year, including stratified by age and sex. Secondary objectives were to examine associations between select characteristics and hospitalization following tSCI, as well as to assess sports-related tSCIs over time, including by individual sport and geographic region.

**Methods:**

We used the Healthcare Cost and Utilization Project Nationwide Emergency Department Sample to identify ED visits among children between January 2016 and December 2020 for incident tSCI. Diagnosis codes were used to identify tSCI and sports-related injury etiologies. Census Bureau data were used to approximate annual rates of pediatric ED visits for tSCI per 100,000 children. Unconditional logistic regression modeling assessed whether select factors were associated with hospital admission.

**Results:**

We found that the annual ED visit rate for tSCI remained relatively stable between 2016 and 2020, with approximately 2,200 new all-cause pediatric ED visits for tSCI annually. Roughly 70% of ED visits for tSCI resulted in hospitalization; most ED visits for tSCI were by older children (15–20 years) and males, who were also more often admitted to the hospital. Notable secondary findings included: (a) compared with older children (15–20 years), younger children (10–14 years) were less likely to be hospitalized immediately following an ED visit for tSCI; (b) patient sex and race were not associated with hospital admission; and (c) American tackle football was the leading cause of sports-related ED visits for tSCI among children. Our findings also suggest that the proportion of sports-related tSCI ED visits may have increased in recent years.

**Discussion:**

Future research should further examine trends in the underlying etiologies of pediatric tSCI, while assessing the effectiveness of new and existing interventions aimed at tSCI prevention.

## Introduction

Traumatic spinal cord injury (tSCI) is a debilitating neurological condition that results in lifelong disability and impairment for many individuals and imposes substantial economic stress on healthcare systems worldwide (1, 2). Individuals with tSCI often have complex healthcare needs, which may be associated with multimorbidity and lower quality of life (3, 4).

Although the incidence of pediatric tSCI in the United States has declined in recent years, there remain limited published data on pediatric emergency department (ED) visits for tSCI and the underlying etiology of these injuries (5–7). One national study from the United States found an average of 1,308 annual ED visits with a principal diagnosis of tSCI for individuals less than 18 years of age between 2007 and 2010 (7). Over 60% of ED visits led to hospitalization; however, 20.1% of children were discharged home (7). A more recent national study from the United States observed that there were over 1,200 tSCI hospitalizations for individuals less than 21 years of age in 2016 (8).

Furthermore, despite the considerable contribution that sports play in the etiology in tSCI, few recent studies have explored the epidemiology of sports-related tSCI in children (less than 21 years of age) (9–11). One study found that while motor vehicle crashes were the most common documented external cause of injury code, sports-related pediatric tSCI were more common among older children (9). Given the impact of sports on tSCI, it may also be important to understand the relationship between tSCI incidence and the reported reduction in physical activity globally during the COVID-19 pandemic (12, 13). Addressing these knowledge gaps is key in injury prevention, as sport-related SCIs are a potential target area for public health education and interventions to improve knowledge and awareness of sports safety.

Therefore, the primary objectives of our study were to describe national trends in incident ED visits for tSCI among children (less than 21 years) between 2016 and 2020 in the United States and to determine the proportion of ED visits that resulted in immediate hospitalization each year, including stratified by age and sex. Our secondary objectives were to examine associations between select characteristics (such as age, sex, and race) and hospitalization following tSCI, as well as to assess sports-related tSCIs over time, including by individual sport and geographic region.

## Materials and methods

### Ethics and reporting

This study was exempt from ethics board review by the Office of Research Ethics at the University of British Columbia. Informed consent was not required from study participants since the data provider deidentified all health records. Furthermore, this research was conducted according to the terms outlined in the United States Agency for Healthcare Research and Quality (AHRQ) Healthcare Cost and Utilization Project (HCUP) Data Use Agreement. This included suppressing small cell counts less than or equal to 10. Our study complies with the Reporting of studies Conducted using Observational Routinely collected Data (RECORD) statement (Supplementary Table 1) (14).

### Data source and study design

Multiple years (2016–2020) of the HCUP Nationwide Emergency Department Sample (NEDS) were used. The NEDS is the largest annual ED database with data for all payers in the United States; it is a stratified probability sample of community, non-rehabilitation, hospital-owned EDs. Approximately 20% of the universe of EDs were sampled within each stratum, with sample weights being computed by HCUP for individual ED discharges and hospitals, respectively (15). Due to its rigorous sampling and weighting strategy, the NEDS is a valuable database that may be used to compute nationally representative estimates of ED visits in the United States. In 2020, the NEDS contains data for more than 28 million distinct ED visits, which, when weighted, are representative of more than 120 million unique ED encounters. Detailed clinical and nonclinical data is recorded in the NEDS for each ED visit and corresponding admission, including but not limited to: International Classification of Diseases, Tenth Revision, Clinical Modification/Procedure Coding System (ICD-10-CM/PCS; beginning 1 October 2015) diagnosis, procedure, and external cause of morbidity codes; patient demographic details (such as age, sex, race, and quartile of median household income); hospital characteristics (such as region, teaching status, trauma center designation, and ownership); and information about healthcare charges (ED charges and, where applicable, inpatient charges) and the payer (such as Medicare, private insurance, or no charge).

### Emergency department visits

Eligible ED visits examined in our study included those where a primary or secondary ICD-10-CM diagnosis of initial traumatic spinal cord injury (tSCI) was recorded among children (less than 21 years).

Study visits were identified using HCUP’s clinical classifications software refined (CCSR, v2022.1) category for “SCI, initial encounter” (INJ009) (16). The CCSR aggregates individual ICD-10-CM codes into more than 530 clinically meaningful categories across 22 body systems. Clinical experts and epidemiologists from our team reviewed ICD-10-CM codes in CCSR category INJ009 to confirm that all available incident tSCI diagnostic codes were included in our algorithm to identify eligible ED visits (8, 17). All diagnostic codes used in our study are provided in Supplementary Tables 2, 3.

### Trend analyses

The annual number of nationwide ED visits for incident tSCI among children between 2016 and 2020 was our primary study outcome, whereas immediate hospital admission, which included admissions to the same hospital and transfers to other short-term hospitals, was our secondary study outcome. Transfers to other short-term hospitals were classified as hospital admissions based on the presumption that the majority of such transfers would result in an inpatient stay. Using HCUP ED discharge weights, we estimated the total national number of pediatric ED visits for tSCI in each calendar year from 2016 to 2020, as well as the total number of visits for tSCI in each year by age (0–4, 5–9, 10–14, and 15–20 years) and sex. We then used yearly US Census Bureau data provided by HCUP to approximate overall annual rates of pediatric ED visits for tSCI per 100,000 children and tSCI visit rates stratified by age and sex.

The number of ED visits for tSCI resulting in immediate hospital admission were examined in each year and reported as a percentage of total ED visits for tSCI. Annual hospital admission percentages were also stratified by age and sex.

### Hospital admissions

Hospital admission analyses were limited to years 2019 and 2020, the last calendar year prior to the COVID-19 pandemic and the first year of the COVID-19 pandemic, respectively. At the time of our study, NEDS data after 2020 was not available. For these analyses, we excluded ED visits where patient payer status, zip income quartile, or race were missing and ED visits where the hospital trauma level designation was unknown.

Descriptive statistics were used to summarize sociodemographic, clinical, and hospital characteristics. Chi-square tests were used to determine whether distributions across examined ED sociodemographic, clinical, and hospital categories differed between children admitted and not admitted to hospital, respectively.

Unconditional logistic regression modeling was used to assess whether select sociodemographic and clinical factors were associated with immediate hospital admission. The same multivariable model was developed for each calendar year. Model covariates were selected *a priori* if they were presumed to be associated with hospital admission and included: age, sex, race, primary payer, and hospital trauma level designation. Models accounted for the complex NEDS survey design by including the strata and clustering of patients and hospitals to compute precise variance estimates for adjusted odds ratios. The significance level was set to 0.05 for all analyses.

### Subgroup analyses: sports-related injuries

Our subgroup analyses focused on ED visits for tSCI that resulted from sports-related injuries. For these analyses, ED visits for tSCI from our primary analyses were queried for recorded diagnoses of 65 distinct sports-related injuries, which were defined using ICD-10-CM codes (Supplementary Table 3) (18, 19). Next, among the subset of ED visits with sports-related injuries, visits were categorized as being attributed to “contact-collision,” “limited contact,” “noncontact,” or “other” sports using the “*Classification of Sports According to Contact*,” which was developed by the American Academy of Pediatrics Council on Sports Medicine and Fitness (18).

We then estimated the total number of ED visits for incident tSCI in each year (2016–2020), the corresponding visit rate per 100,000 children, and the percentage of ED visits leading to immediate hospitalization for the subgroup of pediatric sports-related ED visits. Similar to our primary analyses, reported estimates were stratified by age and sex. Yearly ED visit counts and rates per 100,000 children were also estimated and reported for each sport category.

Lastly, to describe sports most responsible for precipitating tSCI ED visits, all subgroup visits for years 2019 and 2020 were reported in order of decreasing prevalence by individual sport for each year. Annual rankings were further stratified by region (northeast, midwest, south, and west) (20) to characterize geographical differences in sport injury etiology.

### Software

All study analyses were completed using SAS V.9.4 (SAS Institute Inc., Cary, North Carolina, United States). Trends in ED visits were graphically depicted using GraphPad Prism Version 9.2.0 (GraphPad Software LLC, San Diego, California, United States).

## Results

### Trends in emergency department visits

**Figure 1:**
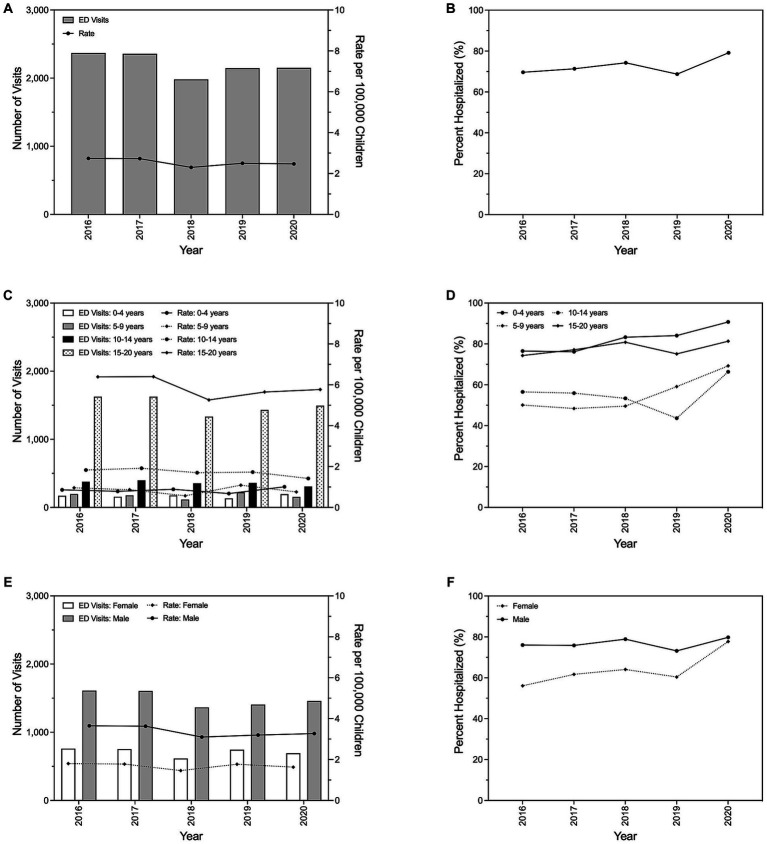
Trends in emergency department visits for pediatric traumatic spinal cord injury and subsequent hospital admissions in the United States, 2016–2020. Total number of visits and corresponding visit rate per year **(A)**, and the proportion of visits resulting in immediate hospitalization by year **(B)**. Annual emergency department visits, visit rate, and proportion of visits resulting in immediate hospitalization stratified by age **(C,D)** and sex **(E,F)**.

Annual trends in pediatric emergency department (ED) discharges for all-cause incident traumatic spinal cord injury (tSCI) between 2016 and 2020 are shown in Figure 1. Between 1 January 2016 and 31 December 2020, there were 11,005 ED visits in the United States for tSCI among children, corresponding to an average of 2,201 visits (standard deviation (SD): ±163) per year. The number of tSCI visits was lowest in 2018 (1,981 visits) and highest in 2016 (2,370 visits). The annual ED visit rate for tSCI remained relatively stable throughout the study period, with 2.74 visits per 100,000 children observed in 2016 and 2.47 visits per 100,000 children observed in 2020 [mean: 2.55 visits per 100,000 children per year, standard deviation (SD): ±0.19 visits per 100,000 children per year], and only decreased by 1.2% between 2019 (2.50 visits per 100,000 children) and 2020, the first year of the COVID-19 pandemic (Figure 1A). Except for 2019 (68.7%), the percentage of pediatric ED visits for tSCI resulting in immediate hospitalization increased yearly between 2016 (69.6%) and 2020 (79.1%; Figure 1B).

Age-stratified trends demonstrated that the oldest children (15–20 years) were consistently responsible for the highest number of tSCI ED visits (mean: 1,501 visits, SD: ±126 visits) and the greatest annual tSCI ED visit rate over time (mean: 5.89 visits per 100,000 children per year, SD: ±0.50 visits per 100,000 children per year; Figures 1C). The average annual tSCI ED visit rate for the oldest children was 243%–597% greater than the average annual visit rate of any other examined age group. Despite the youngest children (0–4 years) having the lowest average annual ED tSCI visit rate (mean: 0.84 visits per 100,000 children per year, SD: ±0.12 visits per 100,000 children per year), they had the highest average annual hospitalization percentage (82.1%; Figure 1D). Their average annual hospitalization percentage most resembled that observed for the oldest children (77.7%). Conversely, the average annual hospitalization percentage for children ages 5–9 years and 10–14 years were similar at 55.3% and 55.2%, respectively.

The number of annual tSCI ED visits (mean: 1,489 visits, SD: ±113 visits) and the corresponding average annual ED tSCI visit rate (mean: 3.37 visits per 100,000 children per year, SD: ±0.25 visits per 100,000 children per year) for males was approximately twice the average number of annual visits (mean: 713 visits, SD: ±60 visits) and visit rate (mean: 1.69 visits per 100,000 children per year, SD: ±0.14 visits per 100,000 children per year), respectively, observed for females. Overall, the annual number of tSCI ED visits and the annual tSCI ED visit rate were relatively stable by sex over time. Although the annual percentage of ED visits resulting in immediate hospitalization was consistently higher for males compared with females, the annual hospitalization percentage for females increased by 38.7% between 2016 (56.1%) and 2020 (77.7%).

### Emergency department visits and hospital admissions

There were 2,146 and 2,151 pediatric ED visits for all-cause tSCI in 2019 and 2020, respectively. A total of 182 (8.5%) and 120 (5.6%) encounters were excluded from our 2019 and 2020 analyses, respectively, due to missing or unknown patient payer status, zip income quartile, race, or hospital trauma level designation. After applying study-specific exclusions, 1,964 and 2,031 pediatric ED visits remained for all-cause tSCI in 2019 and 2020, respectively. The majority (2019: 85.4%; 2020: 83.7%) of ED visits were by older children (10–20 years), while more than two-thirds of visits (2019: 66.5%; 2020: 67.3%) were by males. Most ED visits were by white children (2019: 53.5%; 2020: 49.9%) and private medical insurance (43.3%–45.1%) was the most common primary payer. Cervical injuries were most prevalent (2019: 50.6%; 2020: 46.6%) and few visits to the ED resulted in death (2019: 3.5%; 2020: 1.6%). Most tSCI ED visits occurred in the south (2019: 40.3%; 2020: 42.0%) and midwest (2019: 25.2%; 2020: 24.1%). Children with tSCI mostly presented to level I trauma hospitals (2019: 51.8%; 2020: 58.2%) and hospitals designated as metropolitan teaching centers (2019: 86.4%; 2020: 86.1%). For our admission analyses, 1,321 (67.3%) and 1,605 (79.0%) ED visits resulted in hospital admission in 2019 and 2020, respectively.

Relative to the oldest children (15–20 years), children aged 10–14 years were significantly less likely to be admitted to the hospital immediately following a visit to the ED for tSCI in both 2019 [adjusted odds ratio (AOR) 0.28, 95% CI 0.15 to 0.51] and 2020 (AOR 0.41, 95% CI 0.20 to 0.85). Patient sex and race were not found to be associated with hospital admission. Compared with ED visits covered by private insurance, those subsidized by Medicaid in 2020 were significantly more likely to result in hospital admission (AOR 2.04, 95% CI 1.05 to 3.98). Lastly, non-trauma center ED visits were significantly less likely (2019: AOR 0.25, 95% CI 0.01 to 0.61; 2020: AOR 0.14, 95% CI 0.07 to 0.30) to end in hospital admission than visits to level I trauma centers.

### Trends in sports-related injuries

**Figure 2:**
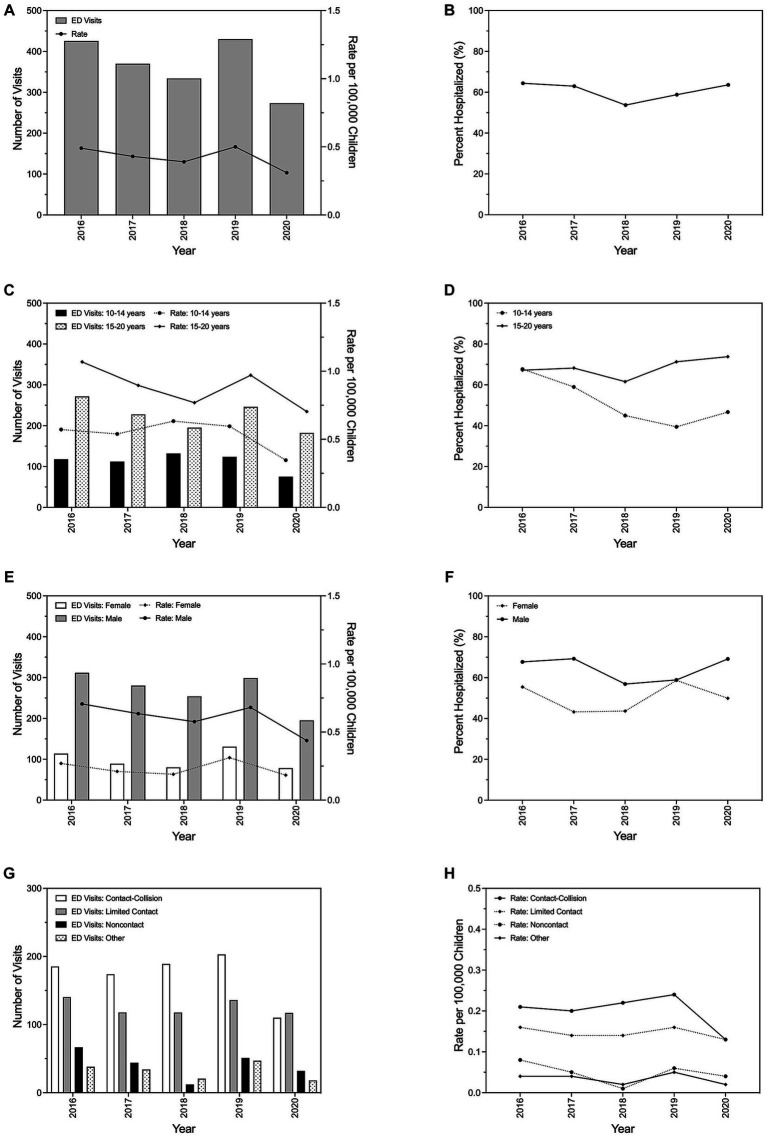
Trends in emergency department visits for pediatric traumatic spinal cord injury resulting from sports-related injuries and subsequent hospital admissions in the United States, 2016–2020. Total number of visits and corresponding visit rate per year **(A)**, and the proportion of visits resulting in immediate hospitalization by year **(B)**. Annual emergency department visits, visit rate, and proportion of visits resulting in immediate hospitalization stratified by age **(C,D)** and sex **(E,F)**. Total number of yearly emergency department visits **(G)** and corresponding annual visit rate **(H)** by sport type.

Compared with all-cause ED tSCI, similar trends in the annual number of tSCI ED visits, tSCI ED visit rates, and immediate hospitalizations were observed for the subgroup of children with sports-related injuries (Figure 2). Annual ED tSCI visits and visit rates attributed to sports-related injuries remained stable between 2016 and 2019 (0.49 visits per 100,000 children in 2016 and 0.50 visits per 100,000 children); however, a 38.0% decrease in the rate was observed between 2019 and 2020 (Figure 2A). The average annual percentage of ED visits for tSCI resulting in immediate hospitalization was lower for sports injuries (60.1%), ranging from 64.4% in 2016 to 63.6% in 2020 (Figure 2B), than was observed for all-cause tSCI (Table 1).

**Table 1:** Baseline characteristics and associations between select factors and immediate hospital admission for pediatric spinal cord injury by year, 2019–2020.

	2019					2020				
		Admitted to hospital					Admitted to hospital			
	All ED visits *n* (%)	Yes *n* (%)	No *n* (%)			All ED visits *n* (%)	Yes *n* (%)	No *n* (%)		
Characteristic	*n* = 1,964	*n* = 1,321	*n* = 643	*p*-values^a^	AOR	*n* = 2,031	*n* = 1,605	*n* = 426	*p*-values^a^	AOR
**Age**										
0–4	113 (5.8)	96 (84.8)	17 (15.2)	<0.01	2.50 (0.88–7.06)	178 (8.8)	164 (92.4)	13 (7.6)	0.01	2.75 (0.78–9.66)
5–9	173 (8.8)	83 (47.9)	90 (52.1)		0.31 (0.12–0.84)^*^	153 (7.5)	106 (69.0)	47 (31.0)		0.40 (0.16–1.04)
10–14	313 (15.9)	124 (39.6)	189 (60.4)		0.28 (0.15–0.51)^***^	295 (14.5)	196 (66.3)	99 (33.7)		0.41 (0.20–0.85)^*^
15–20	1,365 (69.5)	1,018 (74.6)	347 (25.4)		Reference	1,405 (69.2)	1,139 (81.1)	266 (18.9)		Reference
**Sex**										
Male	1,307 (66.5)	945 (72.3)	361 (27.7)	0.01	Reference	1,367 (67.3)	1,090 (79.7)	277 (20.3)	0.60	Reference
Female	657 (33.5)	375 (57.1)	282 (42.9)		0.61 (0.36–1.01)	664 (32.7)	515 (77.5)	149 (22.5)		0.94 (0.56–1.58)
**Race**										
White	1,052 (53.5)	684 (65.1)	367 (34.9)	0.24	Reference	1,013 (49.9)	790 (78.0)	223 (22.0)	0.70	Reference
Black	455 (23.2)	344 (75.6)	111 (24.4)		1.26 (0.62–2.59)	479 (23.6)	372 (77.6)	107 (22.4)		0.53 (0.27–1.04)
Other^b^	457 (23.3)	292 (64.0)	165 (36.0)		1.12 (0.54–2.32)	539 (26.5)	443 (82.2)	96 (17.8)		0.95 (0.45–2.02)
**Primary payer**										
Private insurance	885 (45.1)	621 (70.2)	264 (29.8)	0.62	Reference	880 (43.3)	650 (73.9)	230 (26.1)	0.14	Reference
Medicaid	833 (42.4)	537 (64.4)	296 (35.6)		0.68 (0.35–1.29)	853 (42.0)	713 (83.6)	140 (16.4)		2.04 (1.05–3.98)^*^
Other^c^	246 (12.5)	163 (66.2)	83 (33.8)		0.75 (0.35–1.64)	299 (14.7)	242 (81.2)	56 (18.8)		1.87 (0.77–4.50)
**Median household income** ^**d**^										
Quartile 4	388 (19.8)	213 (55.0)	175 (45.0)	0.40	--	291 (14.3)	212 (73.1)	78 (26.9)	0.75	--
Quartile 3	434 (22.1)	306 (70.5)	128 (29.5)			418 (20.6)	327 (78.4)	90 (21.6)		--
Quartile 2	529 (27.0)	367 (69.3)	163 (30.7)		--	605 (29.8)	477 (78.9)	128 (21.1)		--
Quartile 1	612 (31.2)	434 (71.0)	177 (29.0)		--	718 (35.3)	588 (81.9)	130 (18.1)		--
**Injury level**										
Cervical	993 (50.6)	646 (65.0)	347 (35.0)	0.39	--	947 (46.6)	682 (72.1)	264 (27.9)	0.01	--
Thoracic	670 (34.1)	510 (76.1)	160 (23.9)	0.01	--	729 (35.9)	611 (83.8)	118 (16.2)	0.11	--
Lumbar	332 (16.9)	208 (62.6)	124 (37.4)	0.52	--	464 (22.8)	396 (85.5)	67 (14.5)	0.06	--
Died	68 (3.5)	59 (86.0)	10 (14.0)	0.09	--	32 (1.6)	23 (71.5)	9 (28.5)	0.67	--
**Hospital region**										
Northeast	243 (12.4)	150 (61.7)	93 (38.3)	0.41	--	254 (12.5)	215 (84.6)	39 (15.4)	0.50	--
Midwest	495 (25.2)	353 (71.2)	142 (28.8)		--	489 (24.1)	367 (74.9)	123 (25.1)		--
South	792 (40.3)	573 (72.3)	219 (27.7)		--	853 (42.0)	662 (77.6)	191 (22.4)		--
West	433 (22.1)	245 (56.5)	188 (43.5)		--	435 (21.4)	361 (83.1)	74 (16.9)		--
**Hospital trauma level designation**										
Not a trauma center	262 (13.3)	107 (41.0)	155 (59.0)	<0.01	0.25 (0.10–0.61)^**^	243 (12.0)	109 (45.0)	134 (55.0)	<0.01	0.14 (0.07–0.30)^***^
Trauma center level I	1,017 (51.8)	741 (72.8)	276 (27.2)		Reference	1,182 (58.2)	981 (83.0)	201 (17.0)		Reference
Trauma center level II	533 (27.1)	388 (72.8)	145 (27.2)		0.80 (0.36–1.78)	462 (22.7)	405 (87.7)	57 (12.3)		1.30 (0.60–2.79)
Trauma center level III	152 (7.7)	85 (56.1)	67 (43.9)		0.40 (0.16–1.03)	144 (7.1)	109 (75.7)	35 (24.3)		0.52 (0.18–1.52)
**Teaching status of hospital**										
Metropolitan non-teaching	132 (6.7)	82 (61.7)	51 (38.3)	0.11	--	168 (8.3)	114 (67.9)	54 (32.1)	<0.01	--
Metropolitan teaching	1,696 (86.4)	1,175 (69.3)	522 (30.7)		--	1,748 (86.1)	1,445 (82.7)	303 (17.3)		--
Non-metropolitan hospital	135 (6.9)	64 (47.5)	71 (52.5)		--	116 (5.7)	46 (39.9)	70 (60.1)		--

AOR, adjusted odds ratio; ED, emergency department. ^a^Chi-square test. ^b^Includes Hispanic, Asian or Pacific Islander, Native American, and other races. ^c^Includes Medicare, private insurance, self-pay, and other payers. ^d^2019—Quartile 1: $1–$47,999; Quartile 2: $48,000–$60,999; Quartile 3: $61,000–$81,999; Quartile 4: $82,000+. 2020—Quartile 1: $1–$49,999; Quartile 2: $50,000–$64,999; Quartile 3: $65,000–$85,999; Quartile 4: $86,000+ . ^***^p < 0.001; ^**^p < 0.01; ^*^p < 0.05.

Few young children (0–9 years) visited the ED for tSCI resulting from sports between 2016 and 2020; therefore, associated counts and rates for this population are unable to be reported (Figure 2C). When limited to older children or stratified by sex, annual ED tSCI visits and visit rates for sports-related tSCI minimally fluctuated between 2016 and 2019, though markedly declined in 2020. The average annual ED tSCI visit rate (mean: 0.61 visits per 100,000 children per year, SD: ±0.11 visits per 100,000 children per year) for sports-related injuries among males was nearly three times the visit rate (mean: 0.23 visits per 100,000 children per year, SD: ±0.05 visits per 100,000 children per year) observed for females (Figure 2E).

Between 2016 and 2019, contact-collision and limited contact injuries were the first and second, respectively, causes of sports-related tSCI among children (Figures 2G,H). Although the rate of tSCI ED visits due to contact-collision sports was consistently the highest between 2016 and 2020, the rate sharply declined by 46.5% from 2019 to 2020, matching the rate for limited contact sports injuries (0.13 per 100,000 in 2020).

### Sports-related causes of spinal cord injury

Sports-related injuries accounted for 20.0% of ED visits for tSCI in 2019 (*n* = 2,146), but only 12.7% of similar visits in 2020 (*n* = 2,151). Sports injuries prompting tSCI ED visits among children in 2019 and 2020, including by geographic region, are described in Table 2. The total number of sports-related ED visits for tSCI decreased from 430 encounters in 2019 to 273 encounters in 2020 (36.5% decrease). American tackle football was the leading cause of sports-related tSCI among children in both 2019 and 2020, accounting for 21.1% and 16.9% of tSCI ED visits, respectively. Other sports leading to tSCI in 2019 were varied, with no individual sport accounting for more than 10% of all sports-related tSCI ED visits. Trampolining (8.2%) was second to American tackle football as the most prevalent sports-related injury in 2019. In 2020, snow sports, including skiing (alpine and downhill), snowboarding, sledding, tobogganing, and snow tubing, became the second leading sport cause of tSCI, representing 14.4% of total sports-related injuries. Variations in the prevalence of sports-related injuries were observed by region; however, for the most part, 10 or fewer tSCI ED visits were attributed to individually examined sports within each region.

**Table 2:** Sports-related causes of spinal cord injury by year and region in the United States, 2019–2020.

		Sports-related ED visits for tSCI by region^^^			
2019: sports-related ED visits for tSCI	All regions *n* (%)^^^ (*n* = 430 total)	Northeast (*n* = 80 total)	Midwest (*n* = 97 total)	South (*n* = 135 total)	West (*n* = 118 total)
American tackle football	90 (21.1)	American tackle football (*n* = 16, 20.6%)	American tackle football (*n* = 24, 25.0%)	American tackle football (*n* = 33, 24.4%)	American tackle football (*n* = 17, 14.3%)
Trampolining	35 (8.2)	Ice hockey (*n* = 11, 14.0%)	Wrestling (*n* = 11, 14.3%)	Trampolining (*n* = 16, 11.7%)	Snow sports^+^ (*n* = 14, 11.9%)
^*^Climbing, rappelling and jumping off	33 (7.6)	Basketball^a^	Snow sports^+^	Swimming (*n* = 13, 9.9%)	Soccer^a^
Bike riding	29 (6.7)	Gymnastics^a^	Trampolining	^*^Climbing, rappelling and jumping off (*n* = 13, 9.3%)	Trampolining^a^
Wrestling	26 (6.1)	^*^Climbing, rappelling and jumping off^b^	Bike riding^a^	Wrestling (*n* = 12, 9.1%)	Bike riding^a^
Snow sports^+^	24 (5.6)	Springboard and platform diving^b^	Soccer^a^	Cheerleading^a^	Rugby^a^
Basketball	24 (5.5)	American flag or touch football^c^	^*^Climbing, rappelling and jumping off^b^	Other involving muscle strengthening exercises^a^	^*^Climbing, rappelling and jumping off^b^
Soccer	22 (5.1)	Bike riding^c^	Springboard and platform diving^b^	Basketball^a^	Walking, marching and hiking^b^
Swimming	18 (4.2)	Walking, marching and hiking^c^	Mountain climbing, rock climbing and wall climbing^b^	Bike riding^a^	Gymnastics^c^
Gymnastics	18 (4.1)	Surfing, windsurfing and boogie boarding	Surfing, windsurfing and boogie boarding^c^	Water skiing and wake boarding^b^	Aerobic and step exercise^c^
Springboard and platform diving	16 (3.6)	Running	Basketball^c^	Other specified sports and athletics^b^	Basketball^c^
Walking, marching and hiking	15 (3.4)			Gymnastics^c^	Springboard and platform diving^c^
Ice hockey	11 (2.6)			Soccer^c^	Swimming^c^
Rugby	--			BASE jumping^c^	^#^Other sports and athletics played individually
Surfing, windsurfing and boogie boarding	--			Lacrosse and field hockey^c^	
Running	--			Running^c^	
Cheerleading^a^	--				
Other involving muscle strengthening exercises^a^	--				
American flag or touch football^b^	--				
Water skiing and wake boarding^b^	--				
Mountain climbing, rock climbing and wall climbing^b^	--				
Aerobic and step exercise^b^	--				
Other specified sports and athletics^b^	--				
^#^Other sports and athletics played individually^c^	--				
BASE jumping^c^	--				
Lacrosse and field hockey^c^	--				

2020: sports-related ED visits for tSCI	All regions *n* (%)^^^ (*n* = 273 total)	Northeast (*n* = 53 total)	Midwest (*n* = 73 total)	South (*n* = 105 total)	West (*n* = 42 total)
Percent (%) change from 2019	−36.5	−33.7	−24.7	−22.2	−64.4
American tackle football	46 (16.9)	Snow sports^+^ (*n* = 18, 33.4%)	American tackle football (*n* = 22, 29.9%)	American tackle football (*n* = 20, 18.5%)	Snow sports^+^
Snow sports^+^	39 (14.4)	Ice hockey (*n* = 11, 20.6%)	Snow sports^+^ (*n* = 12, 15.9%)	Swimming (*n* = 17, 16.5%)	Surfing, windsurfing and boogie boarding
Swimming	24 (8.9)	Horseback riding	Basketball	Wrestling	Bike riding^a^
Basketball	17 (6.0)	American tackle football^a^	Bike riding^a^	Springboard and platform diving	Gymnastics^a^
Bike riding	16 (5.9)	Surfing, windsurfing and boogie boarding^a^	Cheerleading^a^	^*^Climbing, rappelling and jumping off^a^	Trampolining^a^
Wrestling	16 (5.7)	Gymnastics	^*^Climbing, rappelling and jumping off^a^	Basketball^a^	Soccer^b^
Springboard and platform diving	14 (5.3)		Springboard and platform diving^a^	Bike riding	Wrestling^b^
^*^Climbing, rappelling and jumping off	14 (5.2)		Ice hockey^b^	^#^Other sports and athletics played individually^b^	Swimming^b^
Ice hockey	14 (5.0)		Swimming^b^	Roller skating (inline) and skateboarding^b^	
Surfing, windsurfing and boogie boarding	11 (3.9)		Wrestling	Other specified sports and athletics^b^	
Horseback riding^a^	--			Cheerleading^b^	
Cheerleading^a^	--			Other involving water and watercraft^b^	
Gymnastics	--			Running^b^	
Trampolining	--			Trampolining^b^	
^#^Other sports and athletics played individually^b^	--			Walking, marching and hiking^b^	
Roller skating (inline) and skateboarding^b^	--				
Other specified sports and athletics^b^	--				
Other involving water and watercraft^b^	--				
Running^b^	--				
Walking, marching and hiking^b^	--				
Soccer^b^	--				

ED, emergency department; tSCI, traumatic spinal cord injury. ^+^Snow (alpine; downhill) skiing, snowboarding, sledding, tobogganing and snow tubing. ^*^Other involving climbing, rappelling and jumping off. ^#^Other involving other sports and athletics played individually. ^^^Reported percentages were based on weighted values and may therefore appear to be inaccurate. ^a^Equal number of visits. ^b^Equal number of visits. ^c^Equal number of visits.

## Discussion

Our primary findings were that the annual ED visit rate for tSCI remained relatively stable between 2016 and 2020, with approximately 2,200 new all-cause pediatric ED visits for tSCI per year. On average, roughly 70% of ED visits for tSCI resulted in immediate hospitalization; most ED visits for tSCI were by older children (15–20 years) and males, who were also more often admitted to the hospital. Notable secondary findings included: (a) compared with older children (15–20 years), younger children (10–14 years) were significantly less likely to be admitted to hospital immediately following a visit to the ED for tSCI; (b) relative to level I trauma centers, ED visits for tSCI at non-trauma centers were significantly less likely to result in hospital admission; (c) patient sex and race were not associated with hospital admission; (d) the proportion of ED visits for tSCI due to sports-related injuries declined between 2019 (20.0%) and 2020 (12.7%); and (f) American tackle football was the leading cause of sports-related ED visits for tSCI among children.

We report that the annual incidence of all-cause ED visits for tSCI among children remained relatively stable between 2016 and 2020 (mean: 2.55 visits per 100,000 children per year), and that the proportion of ED visits for tSCI resulting in immediate hospitalization (mean: 72.6%) marginally increased during the same period (2016: 69.6%; 2020: 79.1%). Despite our study population comprising children to age 20 years, our findings generally coincide with those from a prior NEDS study that examined trends in ED visits for tSCI among children aged 17 years and younger between 2007 and 2010 (7). In that study, investigators determined that an average of 1,308 children and adolescents visited the ED for tSCI each year in the United States, corresponding to a cumulative pediatric tSCI incidence of 1.75 per 100,000 children per year (7). Age and sex disparities in the occurrence of tSCI were reported, whereby ED visits for tSCI were more common among older and male children. Investigators also noted that, overall, 6.9% of tSCIs were attributed to sports and that 62.4% of ED visits for tSCI resulted in admission to the hospital. Compared with our study, observed differences in the cumulative tSCI incidence rate and proportion of sports-related injuries most likely reflect the older age of our study population (68.2% of visits were by children aged 15–20 years) and associated differences in behaviors and lifestyle activities of older children. Other minor differences between study findings may result from variations in case ascertainment algorithms (ICD10-CM vs. ICD-9-CM coding and study-specific primary and secondary tSCI diagnosis inclusion criteria), as well as changes to diagnostic practices in acute clinical settings over time. The elevated proportion of children immediately hospitalized within our study is presumed to be driven by increased tSCI severity among the oldest and youngest children. However, it may also be in part associated with changes in clinical practices, the availability of inpatient beds and specialty care, and health insurance eligibility over the last decade. A separate study using pediatric HCUP inpatient data from 2009 reported that the incidence of tSCI hospitalization among children less than 21 years of age was 2.4 per 100,000 children per year (21), while our prior work using comparable 2016 data from similarly aged children suggested that the incidence of tSCI hospitalization may be as low as 1.48 per 100,000 children per year (8). Compared with our most recent findings, previously observed decreases in the annual pediatric tSCI incidence (6), whether approximated using ED or inpatient encounters, appear to have halted. Additional studies are needed to determine whether further reductions in pediatric tSCI incidence are possible, characterize tSCI etiology and severity further, and inform injury prevention strategies and health resource planning.

Our findings suggest that age and hospital trauma designation may be associated with hospital admission following tSCI among children. Specifically, we observed that children between the ages of 10–14 years were significantly less likely to be admitted to hospital following ED visits for all-cause tSCI compared with the oldest children (15–20 years). This may be due in-part to variations in the mechanisms of tSCI injury, pathology, and level of injury by age. For example, prior studies have repeatedly shown that older children are more likely to sustain sports-related and violent injuries, including those from firearms and assaults, whereas motor vehicle crashes and falls are leading causes of tSCI among younger children (7–10, 22–24). Moreover, we found that ED visits for tSCI occurring at non-trauma centers were significantly less likely to result in hospital admission compared with visits to level I trauma centers. This finding may be explained by the more rural location of select non-trauma centers, differences in distance between patient location where the injury occurred and the nearest hospital, and the often limited capacity of non-trauma centers to respond to severe injuries effectively (25, 26). Although our reported associations are exploratory, they provide valuable insight into factors associated with hospital admission following tSCI, which may in-turn serve as a crude proxy for the quality of tSCI care. Future studies should examine outcomes post-tSCI, including regional differences in inpatient care.

Other studies have repeatedly demonstrated that, following motor vehicle crashes (~37.0%), accidental falls (~20.4%), and firearm injuries (~8.7%), sports (~6.9%) are a major leading cause of tSCI among children (6, 7, 27). Between 2007 and 2010, 6.9% of all ED visits for tSCI among children (0–17 years) in the United States were attributed to sports etiologies (7); whereas between 1997 and 2012, sports injuries were associated with 29.4% and 25.7% of tSCI cases among hospitalizations for spinal injury among children (0–14 years) and adolescents (15–17 years), respectively (6). These observations have led investigators to suggest that pediatric tSCI prevention strategies focused on sports-related injuries may effectively reduce tSCI among children and be more manageable than interventions tailored to other tSCI mechanisms (6). Overall, our findings show that sports-related injuries were documented during 16.6% of all ED visits for tSCI among children between 2016 and 2020; 20.3% and 14.9% of all ED visits for tSCI by children ages 0–14 years and 15–20 years, respectively, were attributed to sports-related causes. We also report that the incidence of pediatric tSCI attributed to sports remained relatively stable between 2016 and 2019. Based on these findings, it is reasonable to hypothesize that reported decreases in pediatric tSCI incidence over the last two decades have been achieved through the positive effects of interventions on injury mechanisms other than sports, such as reductions in tSCI due to motor vehicle crashes (28, 29). Such decreases would explain the decreasing pediatric tSCI incidence over time and the increasing proportion of pediatric tSCI resulting from sports. Notwithstanding, it is also possible that prior approximations of tSCI among children resulting from sports were underestimated due to undocumented mechanisms of injury within health records (7). Irrespective of the causes for the observed shift in the proportion of pediatric sports-related tSCI, further reductions in tSCI incidence may be best attained through investments in public policies, education, and injury prevention initiatives that focus on sports. This may include implementing of broad interventions focused on targeted populations, such as male children or the participants (and their parents) of sports deemed to have an elevated tSCI risk, such as American tackle football, trampolining, or snow sports. It may also include the application of regional risk mitigation strategies in areas with the highest number of pediatric visits to the ED for tSCI, such as the south, or regions with the greatest annual incidence of pediatric sports-related tSCI. Ultimately, further epidemiological investigations into pediatric sport-related tSCI offer the promise of returning considerable public and population health benefits.

Changes in activities of daily living and health behaviors due to COVID-19 restrictions may explain the observed 38.0% decrease in sports-related tSCI between 2019 and 2020 despite the stable overall pediatric tSCI incidence during the same period (13). This is consistent with the reported 15% decrease in the total number of ED visits in the United States from 2019 to 2020. Moreover, the increased 2020 hospital admissions observed in our study correspond with the elevated rate of all-cause admission from the ED reported for the United States in the same year. Future studies will be required to determine how the epidemiology of pediatric tSCI has changed following the first year of the COVID-19 pandemic.

Our study has numerous strengths. Annual pediatric ED visits for tSCI and associated visit rates were estimated using NEDS datasets that are representative of all ED visits in the United States during examined calendar years. Using these datasets allowed us to precisely estimate annual ED visits for tSCI across the 5 years following the introduction of ICD-10-CM coding, including throughout the first year of the COVID-19 pandemic. The identification of eligible tSCI encounters and the classification of sport-related injuries were based on ICD-10-CM detection and categorization algorithms developed in consultation with epidemiologist and clinical experts. Additionally, the datasets included detailed sociodemographic, clinical, and care setting information, which permitted us to: examine trends in ED visits for tSCI by age, sex, and sports-related injuries; characterize immediate hospitalizations following tSCI; and account for factors presumed to confound modeled associations in our regression analyses. To our knowledge, our study is the first to report data from the last decade on trends in national ED visits for tSCI and associated hospital admissions among children in the United States. Therefore, our findings provide meaningful benchmark data that may be used to examine future temporal changes in ED visits for tSCI and assess the effectiveness of interventions targeted at reducing tSCI among children.

Certain limitations should be considered when interpreting our findings. Reported tSCI incidence rates within our study are derived using information from administrative data; they are encounter-based, lack unique patient identifiers, and do not account for children who died at a trauma scene (30). Similar to other studies using electronic health data, it is possible that diagnoses of tSCI, important details pertaining to injury mechanism, and other clinical characteristics were omitted from the administrative datasets or inadvertently misclassified as something else. Such occurrences would have led to the over or underestimation of reported counts and rates within our study and may bias reported estimates of association. Moreover, due to the unavailability of pediatric census data by broad geographic region, we were unable to compute estimates of annual ED visits for tSCI by region. This limited our ability to make specific regional tSCI prevention recommendations, especially regarding preventable sports injuries. Our reported multivariable models are exploratory; we therefore did not make adjustments for multiple comparisons. The models may also not account for all potential confounders that may bias examined associations, such as tSCI severity and household socioeconomic status. Despite these limitations, our reported findings meaningfully address major gaps in the pediatric tSCI literature by advancing existing knowledge of pediatric tSCI incidence and sports-related causes of tSCI.

Overall, we found that the rate of ED visits for pediatric tSCI in the United States was relatively stable between 2016 and 2020, with approximately 2,200 incident cases annually. On average, only approximately 70% of ED visits for tSCI resulted in immediate hospitalization. Future studies are necessary to describe the sociodemographic and clinical characteristics of children that are not hospitalized after visiting the ED for tSCI. Our findings also suggest that the proportion of sports-related tSCI ED visits may have increased in recent years. American tackle football, trampolining, and snow sports were leading causes of sports-related tSCI. Future research should further examine trends in the underlying etiologies of pediatric tSCI, while assessing the effectiveness of new and existing interventions aimed at tSCI prevention.

## Data availability statement

The original contributions presented in the study are included in the article/Supplementary material, further inquiries can be directed to the corresponding author.

## Ethics statement

Ethical approval was not required for the study involving humans in accordance with the local legislation and institutional requirements. Written informed consent to participate in this study was not required from the participants or the participants’ legal guardians/next of kin in accordance with the national legislation and the institutional requirements.

## Author contributions

JCri: Conceptualization, Data curation, Formal analysis, Investigation, Methodology, Project administration, Software, Supervision, Validation, Visualization, Writing – original draft, Writing – review & editing, Funding acquisition. LL: Conceptualization, Writing – original draft, Methodology, Visualization, Formal analysis. VN: Writing – review & editing, Conceptualization. NT: Writing – review & editing, Conceptualization. BK: Writing – review & editing. MD: Writing – review & editing. DT: Writing – review & editing, Conceptualization, Formal analysis, Methodology. AW: Writing – review & editing, Conceptualization, Methodology. JCra: Writing – review & editing, Conceptualization, Formal analysis, Project administration, Supervision.
