# Supplementary material for: Nationwide emergency department visits for pediatric traumatic spinal cord injury in the United States, 2016–2020
Source: Front Neurol. 2023 Nov 10;14:1264589. doi: 10.3389/fneur.2023.1264589 (PMC10667469; doi:10.3389/fneur.2023.1264589)
Supplement: Supplementary file 1 [file Data_Sheet_1.DOCX]

**SUPPLEMENTARY MATERIAL**

**Supplementary Table 1**. The RECORD statement: Checklist of items, extended from the STROBE statement that should be reported in observational studies using routinely collected health data.

| **Section** | **Item Number** | **STROBE Items** | **Location in Article Where Items Are Reported** | **RECORDS Items** | **Location in Article Where Items Are Reported** |
| --- | --- | --- | --- | --- | --- |
| **Title and Abstract** | | | | | |
|  | 1 | (a) Indicate the study’s design with a commonly used term in the title or the abstract. (b) Provide in the abstract an informative and balanced summary of what was done and what was found. | Abstract | RECORD 1.1: The type of data used should be specified in the title or abstract. When possible, the name of the databases used should be included.   RECORD 1.2: If applicable, the geographic region and time frame within which the study took place should be reported in the title or abstract.   RECORD 1.3: If linkage between databases was conducted for the study, this should be clearly stated in the title or abstract. | Abstract (individual databases are also described in the methods section) |
| **Introduction** | | | | | |
| Background rationale | 2 | Explain the scientific background and rationale for the investigation being reported. | Introduction |  |  |
| Objectives | 3 | State specific objectives, including any prespecified hypotheses. | Abstract and Introduction |  |  |
| **Methods** | | | | | |
| Study design | 4 | Present key elements of study design early in the paper. | Methods |  |  |
| Setting | 5 | Describe the setting, locations, and relevant dates, including periods of recruitment, exposure, follow-up, and data collection. | Methods |  |  |
| Participants | 6 | (a) Cohort study: Give the eligibility criteria and the sources and methods of selection of participants. Describe methods of follow-up. Case-control study: Give the eligibility criteria and the sources and methods of case ascertainment and control selection. Give the rationale for the choice of cases and controls. Cross-sectional study: Give the eligibility criteria and the sources and methods of selection of participants. (b) Cohort study: For matched studies, give matching criteria and number of exposed and unexposed. Case-control study: For matched studies, give matching criteria and the number of controls per case. | Methods | RECORD 6.1: The methods of study population selection (such as codes or algorithms used to identify subjects) should be listed in detail. If this is not possible, an explanation should be provided. RECORD 6.2: Any validation studies of the codes or algorithms used to select the population should be referenced. If validation was conducted for this study and not published elsewhere, detailed methods and results should be provided. RECORD 6.3: If the study involved linkage of databases, consider use of a flow diagram or other graphical display to demonstrate the data linkage process, including the number of individuals with linked data at each stage. | Supplementary Tables 2 and 3, Methods, Discussion |
| Variables | 7 | Clearly define all outcomes, exposures, predictors, potential confounders, and effect modifiers. Give diagnostic criteria, if applicable. | Methods | RECORD 7.1: A complete list of codes and algorithms used to classify exposures, outcomes, confounders, and effect modifiers should be provided. If these cannot be reported, an explanation should be provided. | Supplementary Tables 2 and 3 |
| Data sources / measurement | 8 | For each variable of interest, give sources of data and details of methods of assessment (measurement). Describe comparability of assessment methods if there is more than one group. | Methods |  |  |
| Bias | 9 | Describe any efforts to address potential sources of bias. | Methods and Discussion |  |  |
| Study size | 10 | Explain how the study size was arrived at. | Methods |  |  |
| Quantitative variables | 11 | Explain how quantitative variables were handled in the analyses. If applicable, describe which groupings were chosen and why. | Methods |  |  |
| Statistical methods | 12 | 1. Describe all statistical methods, including those used to control for confounding. (b) Describe any methods used to examine subgroups and interactions. (c) Explain how missing data were addressed. (d) Cohort study: If applicable, explain how loss to follow-up was addressed. Case-control study: If applicable, explain how matching of cases and controls was addressed. Cross-sectional study: If applicable, describe analytical methods taking account of sampling strategy. (e) Describe any sensitivity analyses. | (a) Methods  (b) Methods  (c) Methods  (d) Methods  (e) N/A |  |  |
| Data access and cleaning methods |  | N/A |  | RECORD 12.1: Authors should describe the extent to which the investigators had access to the database population used to create the study population. RECORD 12.2: Authors should provide information on the data cleaning methods used in the study. | Methods, Data Sharing Statement |
| Linkage |  | N/A |  | RECORD 12.3: State whether the study included person- level, institutional-level, or other data linkage across two or more databases. The methods of linkage and methods of linkage quality evaluation should be provided. | Methods |
| **Results** | | | | | |
| Participants | 13 | (a) Report the numbers of individuals at each stage of the study (e.g., numbers potentially eligible, examined for eligibility, confirmed eligible, included in the study, completing follow-up, and analysed). (b) Give reasons for nonparticipation at each stage. (c) Consider use of a flow diagram. | Results | RECORD 13.1: Describe in detail the selection of the persons included in the study (i.e., study population selection), including filtering based on data quality, data availability, and linkage. The selection of included persons can be described in the text and/or by means of the study flow diagram. | Results |
| Descriptive data | 14 | 1. Give characteristics of study participants (e.g., demographic, clinical, and social) and information on exposures and potential confounders. (b) Indicate the number of participants with missing data for each variable of interest. (c) Cohort study: summarise follow-up time (e.g., average and total amount). | Results, Table 1 |  |  |
| Outcome data | 15 | Cohort study: Report numbers of outcome events or summary measures over time. Case-control study: Report  numbers in each exposure category or summary measures of exposure. Cross-sectional study: Report numbers of outcome events or summary measures. | Results, Figure 1, Table 1, Figure 2, Table 2 |  |  |
| Main results | 16 | (a) Give unadjusted estimates and, if applicable, confounder-adjusted estimates and their precision (e.g., 95% confidence interval). Make clear which confounders were adjusted for and why they were included. (b) Report category boundaries when continuous variables were categorized. (c) If relevant, consider translating estimates of relative risk into absolute risk for a meaningful time period | Results, Figure 1, Table 1 |  |  |
| Other analyses | 17 | Report other analyses done—e.g., analyses of subgroups and interactions and sensitivity analyses | Results, Figure 2, Table 2 |  |  |
| **Discussion** | | | | | |
| Key results | 18 | Summarise key results with reference to study objectives. | Discussion |  |  |
| Limitations | 19 | Discuss limitations of the study, taking into account sources of potential bias or imprecision. Discuss both direction and magnitude of any potential bias. | Discussion | RECORD 19.1: Discuss the implications of using data that were not created or collected to answer the specific research question(s). Include discussion of misclassification bias, unmeasured confounding, missing data, and changing eligibility over time, as they pertain to the study being reported. | Discussion |
| Interpretation | 20 | Give a cautious overall interpretation of results considering objectives, limitations, multiplicity of analyses, results from  similar studies, and other relevant evidence. | Discussion |  |  |
| Generalizability | 21 | Discuss the generalisability (external validity) of the study results. | Discussion |  |  |
| **Other Information** | | | | | |
| Funding | 22 | Give the source of funding and the role of the funders for the present study and, if applicable, for the original study  on which the present article is based. | Funding acknowledgements |  |  |
| Accessibility of protocol, raw data, and programming code |  | N/A |  | RECORD 22.1: Authors should provide information on how to access any supplemental information such as the study protocol, raw data, or programming code. | Data Sharing Statement |

**Supplementary Table 2.** ICD-10-CM codes used to identify emergency department visits for traumatic spinal cord injury.

| **ICD-10-CM Code** | **ICD-10-CM Code Description** | **CCSR Category** | **Injury location** |
| --- | --- | --- | --- |
| S140XXA | Concussion and edema of cervical spinal cord, initial encounter | INJ009 | Cervical |
| S14101A | Unspecified injury at C1 level of cervical spinal cord, initial encounter | INJ009 | Cervical |
| S14102A | Unspecified injury at C2 level of cervical spinal cord, initial encounter | INJ009 | Cervical |
| S14103A | Unspecified injury at C3 level of cervical spinal cord, initial encounter | INJ009 | Cervical |
| S14104A | Unspecified injury at C4 level of cervical spinal cord, initial encounter | INJ009 | Cervical |
| S14105A | Unspecified injury at C5 level of cervical spinal cord, initial encounter | INJ009 | Cervical |
| S14106A | Unspecified injury at C6 level of cervical spinal cord, initial encounter | INJ009 | Cervical |
| S14107A | Unspecified injury at C7 level of cervical spinal cord, initial encounter | INJ009 | Cervical |
| S14108A | Unspecified injury at C8 level of cervical spinal cord, initial encounter | INJ009 | Cervical |
| S14109A | Unspecified injury at unspecified level of cervical spinal cord, initial encounter | INJ009 | Cervical |
| S14111A | Complete lesion at C1 level of cervical spinal cord, initial encounter | INJ009 | Cervical |
| S14112A | Complete lesion at C2 level of cervical spinal cord, initial encounter | INJ009 | Cervical |
| S14113A | Complete lesion at C3 level of cervical spinal cord, initial encounter | INJ009 | Cervical |
| S14114A | Complete lesion at C4 level of cervical spinal cord, initial encounter | INJ009 | Cervical |
| S14115A | Complete lesion at C5 level of cervical spinal cord, initial encounter | INJ009 | Cervical |
| S14116A | Complete lesion at C6 level of cervical spinal cord, initial encounter | INJ009 | Cervical |
| S14117A | Complete lesion at C7 level of cervical spinal cord, initial encounter | INJ009 | Cervical |
| S14118A | Complete lesion at C8 level of cervical spinal cord, initial encounter | INJ009 | Cervical |
| S14119A | Complete lesion at unspecified level of cervical spinal cord, initial encounter | INJ009 | Cervical |
| S14121A | Central cord syndrome at C1 level of cervical spinal cord, initial encounter | INJ009 | Cervical |
| S14122A | Central cord syndrome at C2 level of cervical spinal cord, initial encounter | INJ009 | Cervical |
| S14123A | Central cord syndrome at C3 level of cervical spinal cord, initial encounter | INJ009 | Cervical |
| S14124A | Central cord syndrome at C4 level of cervical spinal cord, initial encounter | INJ009 | Cervical |
| S14125A | Central cord syndrome at C5 level of cervical spinal cord, initial encounter | INJ009 | Cervical |
| S14126A | Central cord syndrome at C6 level of cervical spinal cord, initial encounter | INJ009 | Cervical |
| S14127A | Central cord syndrome at C7 level of cervical spinal cord, initial encounter | INJ009 | Cervical |
| S14128A | Central cord syndrome at C8 level of cervical spinal cord, initial encounter | INJ009 | Cervical |
| S14129A | Central cord syndrome at unspecified level of cervical spinal cord, initial encounter | INJ009 | Cervical |
| S14131A | Anterior cord syndrome at C1 level of cervical spinal cord, initial encounter | INJ009 | Cervical |
| S14132A | Anterior cord syndrome at C2 level of cervical spinal cord, initial encounter | INJ009 | Cervical |
| S14133A | Anterior cord syndrome at C3 level of cervical spinal cord, initial encounter | INJ009 | Cervical |
| S14134A | Anterior cord syndrome at C4 level of cervical spinal cord, initial encounter | INJ009 | Cervical |
| S14135A | Anterior cord syndrome at C5 level of cervical spinal cord, initial encounter | INJ009 | Cervical |
| S14136A | Anterior cord syndrome at C6 level of cervical spinal cord, initial encounter | INJ009 | Cervical |
| S14137A | Anterior cord syndrome at C7 level of cervical spinal cord, initial encounter | INJ009 | Cervical |
| S14138A | Anterior cord syndrome at C8 level of cervical spinal cord, initial encounter | INJ009 | Cervical |
| S14139A | Anterior cord syndrome at unspecified level of cervical spinal cord, initial encounter | INJ009 | Cervical |
| S14141A | Brown-Sequard syndrome at C1 level of cervical spinal cord, initial encounter | INJ009 | Cervical |
| S14142A | Brown-Sequard syndrome at C2 level of cervical spinal cord, initial encounter | INJ009 | Cervical |
| S14143A | Brown-Sequard syndrome at C3 level of cervical spinal cord, initial encounter | INJ009 | Cervical |
| S14144A | Brown-Sequard syndrome at C4 level of cervical spinal cord, initial encounter | INJ009 | Cervical |
| S14145A | Brown-Sequard syndrome at C5 level of cervical spinal cord, initial encounter | INJ009 | Cervical |
| S14146A | Brown-Sequard syndrome at C6 level of cervical spinal cord, initial encounter | INJ009 | Cervical |
| S14147A | Brown-Sequard syndrome at C7 level of cervical spinal cord, initial encounter | INJ009 | Cervical |
| S14148A | Brown-Sequard syndrome at C8 level of cervical spinal cord, initial encounter | INJ009 | Cervical |
| S14149A | Brown-Sequard syndrome at unspecified level of cervical spinal cord, initial encounter | INJ009 | Cervical |
| S14151A | Other incomplete lesion at C1 level of cervical spinal cord, initial encounter | INJ009 | Cervical |
| S14152A | Other incomplete lesion at C2 level of cervical spinal cord, initial encounter | INJ009 | Cervical |
| S14153A | Other incomplete lesion at C3 level of cervical spinal cord, initial encounter | INJ009 | Cervical |
| S14154A | Other incomplete lesion at C4 level of cervical spinal cord, initial encounter | INJ009 | Cervical |
| S14155A | Other incomplete lesion at C5 level of cervical spinal cord, initial encounter | INJ009 | Cervical |
| S14156A | Other incomplete lesion at C6 level of cervical spinal cord, initial encounter | INJ009 | Cervical |
| S14157A | Other incomplete lesion at C7 level of cervical spinal cord, initial encounter | INJ009 | Cervical |
| S14158A | Other incomplete lesion at C8 level of cervical spinal cord, initial encounter | INJ009 | Cervical |
| S14159A | Other incomplete lesion at unspecified level of cervical spinal cord, initial encounter | INJ009 | Cervical |
| S240XXA | Concussion and edema of thoracic spinal cord, initial encounter | INJ009 | Thoracic |
| S24101A | Unspecified injury at T1 level of thoracic spinal cord, initial encounter | INJ009 | Thoracic |
| S24102A | Unspecified injury at T2-T6 level of thoracic spinal cord, initial encounter | INJ009 | Thoracic |
| S24103A | Unspecified injury at T7-T10 level of thoracic spinal cord, initial encounter | INJ009 | Thoracic |
| S24104A | Unspecified injury at T11-T12 level of thoracic spinal cord, initial encounter | INJ009 | Thoracic |
| S24109A | Unspecified injury at unspecified level of thoracic spinal cord, initial encounter | INJ009 | Thoracic |
| S24111A | Complete lesion at T1 level of thoracic spinal cord, initial encounter | INJ009 | Thoracic |
| S24112A | Complete lesion at T2-T6 level of thoracic spinal cord, initial encounter | INJ009 | Thoracic |
| S24113A | Complete lesion at T7-T10 level of thoracic spinal cord, initial encounter | INJ009 | Thoracic |
| S24114A | Complete lesion at T11-T12 level of thoracic spinal cord, initial encounter | INJ009 | Thoracic |
| S24119A | Complete lesion at unspecified level of thoracic spinal cord, initial encounter | INJ009 | Thoracic |
| S24131A | Anterior cord syndrome at T1 level of thoracic spinal cord, initial encounter | INJ009 | Thoracic |
| S24132A | Anterior cord syndrome at T2-T6 level of thoracic spinal cord, initial encounter | INJ009 | Thoracic |
| S24133A | Anterior cord syndrome at T7-T10 level of thoracic spinal cord, initial encounter | INJ009 | Thoracic |
| S24134A | Anterior cord syndrome at T11-T12 level of thoracic spinal cord, initial encounter | INJ009 | Thoracic |
| S24139A | Anterior cord syndrome at unspecified level of thoracic spinal cord, initial encounter | INJ009 | Thoracic |
| S24141A | Brown-Sequard syndrome at T1 level of thoracic spinal cord, initial encounter | INJ009 | Thoracic |
| S24142A | Brown-Sequard syndrome at T2-T6 level of thoracic spinal cord, initial encounter | INJ009 | Thoracic |
| S24143A | Brown-Sequard syndrome at T7-T10 level of thoracic spinal cord, initial encounter | INJ009 | Thoracic |
| S24144A | Brown-Sequard syndrome at T11-T12 level of thoracic spinal cord, initial encounter | INJ009 | Thoracic |
| S24149A | Brown-Sequard syndrome at unspecified level of thoracic spinal cord, initial encounter | INJ009 | Thoracic |
| S24151A | Other incomplete lesion at T1 level of thoracic spinal cord, initial encounter | INJ009 | Thoracic |
| S24152A | Other incomplete lesion at T2-T6 level of thoracic spinal cord, initial encounter | INJ009 | Thoracic |
| S24153A | Other incomplete lesion at T7-T10 level of thoracic spinal cord, initial encounter | INJ009 | Thoracic |
| S24154A | Other incomplete lesion at T11-T12 level of thoracic spinal cord, initial encounter | INJ009 | Thoracic |
| S24159A | Other incomplete lesion at unspecified level of thoracic spinal cord, initial encounter | INJ009 | Thoracic |
| S3401XA | Concussion and edema of lumbar spinal cord, initial encounter | INJ009 | Lumbar |
| S3402XA | Concussion and edema of sacral spinal cord, initial encounter | INJ009 | Sacral |
| S34101A | Unspecified injury to L1 level of lumbar spinal cord, initial encounter | INJ009 | Lumbar |
| S34102A | Unspecified injury to L2 level of lumbar spinal cord, initial encounter | INJ009 | Lumbar |
| S34103A | Unspecified injury to L3 level of lumbar spinal cord, initial encounter | INJ009 | Lumbar |
| S34104A | Unspecified injury to L4 level of lumbar spinal cord, initial encounter | INJ009 | Lumbar |
| S34105A | Unspecified injury to L5 level of lumbar spinal cord, initial encounter | INJ009 | Lumbar |
| S34109A | Unspecified injury to unspecified level of lumbar spinal cord, initial encounter | INJ009 | Lumbar |
| S34111A | Complete lesion of L1 level of lumbar spinal cord, initial encounter | INJ009 | Lumbar |
| S34112A | Complete lesion of L2 level of lumbar spinal cord, initial encounter | INJ009 | Lumbar |
| S34113A | Complete lesion of L3 level of lumbar spinal cord, initial encounter | INJ009 | Lumbar |
| S34114A | Complete lesion of L4 level of lumbar spinal cord, initial encounter | INJ009 | Lumbar |
| S34115A | Complete lesion of L5 level of lumbar spinal cord, initial encounter | INJ009 | Lumbar |
| S34119A | Complete lesion of unspecified level of lumbar spinal cord, initial encounter | INJ009 | Lumbar |
| S34121A | Incomplete lesion of L1 level of lumbar spinal cord, initial encounter | INJ009 | Lumbar |
| S34122A | Incomplete lesion of L2 level of lumbar spinal cord, initial encounter | INJ009 | Lumbar |
| S34123A | Incomplete lesion of L3 level of lumbar spinal cord, initial encounter | INJ009 | Lumbar |
| S34124A | Incomplete lesion of L4 level of lumbar spinal cord, initial encounter | INJ009 | Lumbar |
| S34125A | Incomplete lesion of L5 level of lumbar spinal cord, initial encounter | INJ009 | Lumbar |
| S34129A | Incomplete lesion of unspecified level of lumbar spinal cord, initial encounter | INJ009 | Lumbar |
| S34131A | Complete lesion of sacral spinal cord, initial encounter | INJ009 | Sacral |
| S34132A | Incomplete lesion of sacral spinal cord, initial encounter | INJ009 | Sacral |
| S34139A | Unspecified injury to sacral spinal cord, initial encounter | INJ009 | Sacral |
| S343XXA | Injury of cauda equina, initial encounter | INJ009 | Cauda Equina |

**Abbreviations:** CCSR, Clinical Classifications Software Refined; ICD-10-CM, International Classification of Diseases, 10th Revision, Clinical Modification.

**Supplementary Table 3.** ICD-10-CM codes used to identify and categorize sports.

| **ICD-10-CM Code** | **ICD-10-CM Code Description** | **Contact type** |
| --- | --- | --- |
| Y9312 | Activity, springboard and platform diving | Contact or collision |
| Y9313 | Activity, water polo | Contact or collision |
| Y9322 | Activity, ice hockey | Contact or collision |
| Y9361 | Activity, American tackle football | Contact or collision |
| Y9363 | Activity, rugby | Contact or collision |
| Y9365 | Activity, lacrosse and field hockey | Contact or collision |
| Y9366 | Activity, soccer | Contact or collision |
| Y9367 | Activity, basketball | Contact or collision |
| Y9371 | Activity, boxing | Contact or collision |
| Y9372 | Activity, wrestling | Contact or collision |
| Y9375 | Activity, martial arts | Contact or collision |
| Y9317 | Activity, water skiing and wake boarding | Limited contact |
| Y9318 | Activity, surfing, windsurfing and boogie boarding | Limited contact |
| Y9319 | Activity, other involving water and watercraft | Limited contact |
| Y9321 | Activity, ice skating | Limited contact |
| Y9323 | Activity, snow (alpine) (downhill) skiing, snowboarding, sledding, tobogganing and snow tubing | Limited contact |
| Y9324 | Activity, cross country skiing | Limited contact |
| Y9329 | Activity, other involving ice and snow | Limited contact |
| Y9343 | Activity, gymnastics | Limited contact |
| Y9344 | Activity, trampolining | Limited contact |
| Y9345 | Activity, cheerleading | Limited contact |
| Y9349 | Activity, other involving dancing and other rhythmic movements | Limited contact |
| Y9351 | Activity, roller skating (inline) and skateboarding | Limited contact |
| Y9352 | Activity, horseback riding | Limited contact |
| Y9355 | Activity, bike riding | Limited contact |
| Y9357 | Activity, non-running track and field events | Limited contact |
| Y9359 | Activity, other involving other sports and athletics played individually | Limited contact |
| Y9362 | Activity, American flag or touch football | Limited contact |
| Y9364 | Activity, baseball | Limited contact |
| Y9368 | Activity, volleyball (beach) (court) | Limited contact |
| Y9369 | Activity, other involving other sports and athletics played as a team or group | Limited contact |
| Y9301 | Activity, walking, marching and hiking | Noncontact/noncollision |
| Y9302 | Activity, running | Noncontact/noncollision |
| Y9311 | Activity, swimming | Noncontact/noncollision |
| Y9314 | Activity, water aerobics and water exercise | Noncontact/noncollision |
| Y9315 | Activity, underwater diving and snorkeling | Noncontact/noncollision |
| Y9316 | Activity, rowing, canoeing, kayaking, rafting and tubing | Noncontact/noncollision |
| Y9341 | Activity, dancing | Noncontact/noncollision |
| Y9342 | Activity, yoga | Noncontact/noncollision |
| Y9353 | Activity, golf | Noncontact/noncollision |
| Y9354 | Activity, bowling | Noncontact/noncollision |
| Y9356 | Activity, jumping rope | Noncontact/noncollision |
| Y936A | Activity, physical games generally associated with school recess, summer camp and children | Noncontact/noncollision |
| Y9373 | Activity, racquet and hand sports | Noncontact/noncollision |
| Y9374 | Activity, frisbee | Noncontact/noncollision |
| Y93A1 | Activity, exercise machines primarily for cardiorespiratory conditioning | Noncontact/noncollision |
| Y93A2 | Activity, calisthenics | Noncontact/noncollision |
| Y93A3 | Activity, aerobic and step exercise | Noncontact/noncollision |
| Y93A4 | Activity, circuit training | Noncontact/noncollision |
| Y93A5 | Activity, obstacle course | Noncontact/noncollision |
| Y93A6 | Activity, grass drills | Noncontact/noncollision |
| Y93A9 | Activity, other involving cardiorespiratory exercise | Noncontact/noncollision |
| Y93B1 | Activity, exercise machines primarily for muscle strengthening | Noncontact/noncollision |
| Y93B2 | Activity, push-ups, pull-ups, sit-up | Noncontact/noncollision |
| Y93B3 | Activity, free weights | Noncontact/noncollision |
| Y93B4 | Activity, pilates | Noncontact/noncollision |
| Y93B9 | Activity, other involving muscle strengthening exercises | Noncontact/noncollision |
| Y9381 | Activity, refereeing a sports activity | Noncontact/noncollision |
| Y9331 | Activity, mountain climbing, rock climbing and wall climbing | Other |
| Y9332 | Activity, rappelling | Other |
| Y9333 | Activity, BASE jumping | Other |
| Y9334 | Activity, bungee jumping | Other |
| Y9335 | Activity, hang gliding | Other |
| Y9339 | Activity, other involving climbing, rappelling and jumping off | Other |
| Y9379 | Activity, other specified sports and athletics | Other |

**Abbreviations:** ICD-10-CM, International Classification of Diseases, 10th Revision, Clinical Modification.
